# Effect of Curing Regime on the Mechanical Strength, Hydration, and Microstructure of Ecological Ultrahigh-Performance Concrete (EUHPC)

**DOI:** 10.3390/ma15051668

**Published:** 2022-02-23

**Authors:** Zhiwu Zuo, Jiachen Zhang, Beixing Li, Chuqi Shen, Gongfeng Xin, Xiao Chen

**Affiliations:** 1 Shandong Key Laboratory of Highway Technology and Safety Assessment, Jinan 250000, China; zzw9000@163.com (Z.Z.); gfxin@163.com (G.X.); 2 Shandong Hi-Speed Group Co., Ltd., Jinan 250000, China; 3 State Key Laboratory of Silicate Materials for Architecture, Wuhan University of Technology, Wuhan 430070, China; 18235123290@139.com (J.Z.); 15236455692@139.com (C.S.); 4 AAC Technologies Holdings Inc., Changzhou 213000, China

**Keywords:** ecological ultrahigh-performance concrete (EUHPC), curing regime, mechanical strength, hydration, microstructure

## Abstract

This paper investigates the effect of curing regimes (standard and steam curing) on the mechanical strength, hydration, and microstructure of ecological ultrahigh-performance concrete (EUHPC). The flowability, compressive strength, flexural strength, hydration, porosity, pore size distribution, and microstructure of UHPC with different contents of supplementary materials (silica fume, fly ash, and ground granulated blast furnace slag) were assessed. The test results showed that the compressive strength of EUHPC under steam curing was increased considerably compared to that under standard curing, while the flexural strength was mildly decreased. The steam curing could decrease the porosity of EUHPC, which ranged between 7% and 9% for standard curing, and between 3.5% and 5% for steam curing. The aperture of EUHPC was below 20 nm, mainly located in the range of 10 nm to 20 nm under standard curing, while it was less than 10 nm for steam curing. C–S–H gel was produced under steam curing, while unhydrated fly ash, mineral powder, and Ca(OH)_2_ crystal were observed in the amorphous C–S–H gel. The microstructure of EUHPC under steam curing was denser than that under standard curing, and the interfacial transition zones under both curing regimes were compact.

## 1. Introduction

Ultrahigh-performance concrete (UHPC) is a novel cement-based composite material, which is characterized by ultrahigh compressive and tensile strength, good ductility due to the addition of steel fiber, and superior durability because of the compact microstructure [1]. To the authors’ knowledge, UHPC was first mentioned in the publication by De Larrard and Sedran [2]. Depending on the mix proportion and the preparation method, the compressive strength of UHPC usually exceeds 150 MPa, with a tensile strength of 6.2 MPa [3,4]. The excellent properties of UHPC are due to the meticulous design of the mix proportion, such as high binder content, low water/binder ratio, and a large dosage of superplasticizer, supplementary materials (silica fume, fly ash, granulated blast furnace slag, etc.), fine quartz powder, and steel fiber. Specifically, the coarse aggregate is usually excluded in most mixtures to increase the homogeneity of matrix, as well as reduce the micro-cracks existing in the interfacial transition zone between the paste matrix and coarse aggregate [4,5,6]. The excellent properties make UHPC an ideal candidate in bridges, high-speed railways, thin-walled structures, shells, repairments, nuclear powder containments, and military engineering [7,8,9,10].

However, the well-chosen raw materials and complicated preparation method limit its extensive use in practical projects due to the low production efficiency and high energy consumption [11]. The cement content in UHPC is usually as high as 900–1100 kg/m^3^ [12,13,14], which not only affects the production costs but also has a significant impact on the hydration heat and may produce severe autogenous shrinkage. Furthermore, it will cause some disadvantages in the modern construction industry, especially in developing countries with a lack of resources and high cost constraints. Furthermore, the world has placed higher requirements on carbon emissions, with China committing to achieve carbon compliance by 2030 and carbon neutrality by 2060 [15]. As commonly known, the production of cement is said to account for about 7% of total carbon emissions [16,17]; hence, resource shortages and environmental requirements induce an urgent need for alternative methods to prepare UHPC with a low environmental footprint. Furthermore, silica fume is indispensable in the mixture design of UHPC, which accounts for 15–30% of the binder to achieve high compressive strength and durability [18,19]. Silica fume has a smaller particle size, which fills the voids in the matrix and accelerates the pozzolanic reaction to produce additional calcium silicate hydrates (C–S–H) [16,20,21,22]. However, the improved performance of UHPC upon adding silica fume is concomitant with an increase in cost, as the price of silica fume is as much as 4–7 times that of Portland cement.

Some researchers have tried to adopt various alternatives, especially industrial co-products, for a high dosage of cement and silica fume to develop ecological ultrahigh-performance concrete (EUHPC), which can reduce the material cost and high autogenous shrinkage of UHPC [4,23,24,25]. For instance, Omar et al. [24] found that the workability, compressive strength, and packing density of UHPC can be significantly improved by replacing 30% cement with blast furnace slag (BBS), whereby the cement hydration and pozzolanic reaction of mineral admixtures are accelerated. Chen et al. [26] observed that the incorporation of fly ash can reduce the porosity of UHPC samples. Aldahdooh et al. [27] prepared green ultrahigh-performance fiber-reinforced cementitious composites (GUHPFRCCs), where 50% of the total binder by the volume fraction was replaced by ultrafine palm oil fuel ash, achieving a 90 days compressive strength, flexural strength, and direct tensile strength of 158.28 MPa, 46.69 MPa, and 13.78 MPa, respectively. Wu et al. [25] found that there is an optimal GGBS (40%) or fly ash (20%) content for the flexural properties of UHPC. Huang et al. [28] and Nguyen et al. [12] incorporated rice husk ash (RHA) in the UHPC mixture to replace silica fume by weight to a certain content, and they reported that the addition of RHA enhances the compressive strength and impermeability of UHPC.

The curing temperature has a significant effect on the mechanical properties of concrete, which can affect the cement hydration and pozzolanic effect of supplementary materials, further influencing the formation of calcium silica hydrate (C–S–H) and the evolution of the microstructure of concrete. Heat treatments have generally been used for UHPC preparation to accelerate the cement hydration and pozzolanic effect of mineral admixtures for improving the strength, density, and microstructure [29,30,31]. However, the heat treatment is limited when cast in place, while the temperature and the moisture are difficult to control at a construction site. Hence, it is important to study the effect of curing regimes on the mechanical properties, hydration, and microstructure development of UHPC. Yazici et al. [32] and Shen et al. [30] reported that the compressive strength was significantly improved after steam and autoclaving, while the flexural strength and toughness were decreased. Wu et al. [25] reported that GGBS or fly ash has a limited effect on the compressive strength of UHPC under standard curing, while the compressive strength decreased with the increase in GGBS and fly ash content under hot water and steam curing. Yang et al. [33] studied the effect of aggregate and curing regime on the mechanical properties of ultrahigh-performance fiber-reinforced concrete (UHPFRC); the experimental results indicated that the compressive strength, flexural strength, and fracture energy under 20 °C curing were 20%, 10%, and 15% lower than those under 90 °C curing, respectively. So far, extensive research on the mechanical and shrinkage properties of UHPC has been conducted considering the effects of supplementary materials (content and type), as well as the curing regimes, but limited information has been published about the effect of supplementary materials on the hydration, porosity, and microstructure development of UHPC under different curing regimes.

The objective of this study was to investigate the effect of curing regime on the mechanical properties, hydration, pore size distribution, and microstructure of EUHPC with different contents of cementitious materials. Two curing regimes and three types of supplementary materials (silica fume, fly ash, and ground granulated blast furnace slag) were considered. The fresh and hardened behaviors of EUHPC were evaluated. Techniques such as X-ray diffraction, mercury injection porosimetry, and scanning electron microscopy were employed to assess the hydration, pore size distribution, and microstructure development of EUHPC.

## 2. Experimental Program

### 2.1. Materials and Mix Proportion

Portland cement with a grade of P.O 52.5 was used as the cementitious material. To reduce the cement usage in the UHPC mixture, three types of supplementary materials by different mass fractions of cementitious materials were used in this study: silica fume (SF), fly ash (FA), and ground granulated blast furnace slag (GGBFS). The major chemical components of cement, silica fume, fly ash, and slag tested by X-ray fluorescence (XRF) (PANalytical. B.V., Almelo, The Netherlands) are summarized in Table 1. Three dosages by the total weight of cementitious materials were considered for SF, FA, and GGBFS. Quartz sand with a size in the range of 0.5 to 1.2 mm was used as the fine aggregate. Coarse aggregate was removed to guarantee the compaction of EUHPC. The aggregate/binder ratio was kept at a constant of 1.0 for all mixtures. Copper-coated straight steel fibers with a length of 13 mm and diameter of 0.2 mm were employed at 2% by volume of the total mixture; the fibers had a tensile strength greater than 2200 MPa. The content of steel fiber was constant for all mixtures. The water–binder ratios of all mixtures were kept at a constant of 0.16. Due to the low W/B ratio, the defoamer and rheology modifier were adopted to improve the flowability of EUHPC at the total mass of 0.1% of the cementitious materials. The detailed mixture proportions of EUHPC are listed in Table 2. The abbreviations of mixtures depend on the type of supplementary material and the ratio by the binding material weight; for instance, ‘SF15FA15G15′ denotes that the ratios by the binding material weight of SF, FA, and GGBFS were 15%. On the other hand, the S20F0G0 mixture constituting 80% cement and 20% silica fume by mass fraction was taken as the reference mixture. In total, eight EUHPC mixtures were prepared.

### 2.2. Specimen Preparation

According to the EUHPC mix proportion, the solid components including the cement, supplementary materials, and fine aggregate were weighted and incorporated in a vertical planetary mixer for 1–2 min. Secondly, the water reducer was uniformly dissolved in water and then added to the dry ingredients to mix for about 5 min at high speed until good flowability was obtained. Thirdly, the steel fibers were dispersed evenly in the mortar, followed by additional mixing for about 5 min until a good flowability and an even distribution of steel fiber in the cement matrix were achieved. After mixing, the fresh EUHPC was poured into plastic molds and compacted in a high-frequency vibrator to remove bubbles and holes in the concrete. After the molding of EUHPC, the surface was covered by a plastic sheet to prevent moisture volatilization during hardening. Prismatic specimens with dimensions of 40 mm × 40 mm × 160 mm were prepared to determine the compressive strength and flexural strength. At least three replicated specimens were prepared for each test.

The specimens were kept in the plastic molds at a room temperature of about 20 °C for 24 h. After demolding, the specimens were cured in a standard curing chamber at 20 ± 2 °C with a humidity of 95% for 7 days. Another batch of specimens was demolded and moved to the steam curing tank, where the heating rate was less than 12 °C/h, and then all specimens were exposed to steam curing at 70 °C for 72 h. Thereafter, the specimens were cooled at a cooling rate of no more than 15 °C/h. After the temperature of the steam curing tank decreased to ambient temperature, the specimens were moved to the standard curing room for 7 days at 20 °C until testing.

### 2.3. Testing Methods

#### 2.3.1. Flowability

After mixing the paste, the flowability test of EUHPC was conducted in accordance with GB 2419-2005 [34] using a flow table (Wuxi Jianyi Experimental Equipment Co., Ltd, Wuxi, China); the fresh EUHPC was poured in a mold and inserted using a tamp. The mold was then removed by opening the jumping table, allowing the paste to spread on the table. After 30 jumps in 30 ± 1 s, the spread was measured.

#### 2.3.2. Mechanical Strength

The flexural and compressive tests were carried out using a 300 kN testing machine (Wuxi Jianyi Experimental Equipment Co., Ltd, Wuxi, China), after which the flexural specimens were tested under four-point loading using displacement control at a loading rate of 0.1 mm/s according to GB 17671-2020 [35]. The loading was terminated upon recording deflection up to 3 mm. The clear span between the simple supports was 130 mm. After the flexural test, the two broken pieces left from the original prism were used for the compressive strength test. The loaded area of the compressive specimen was 40 × 40 mm, and the height was also 40 mm. The loading rate of the compressive test was maintained at 2.4 kN/s until failure.

#### 2.3.3. X-ray Diffraction

The hydration degree of cement was estimated by X-ray diffraction (XRD) (PANalytical. B.V., Almelo, The Netherlands). The small fragments after the compression test were ground into powder and then stored in ethanol to terminate the hydration of cement.

#### 2.3.4. Mercury Injection Porosimetry

The total porosity and pore size distribution of EUHPC samples were tested via mercury injection porosimetry (MIP) (Shenzhen Huapu General Technology Co., Ltd, Shenzhen, China). The samples were taken from the small fragments after the compression test, and two samples with a maximum size of no more than 10 mm were prepared for each mixture. The hydration reaction of EUHPC samples was terminated by immersing the samples in 99.8% isopropyl alcohol. Later, these fragments were dried in a vacuum-drying oven at 105 °C for 12 h to remove the free water before the MIP test. The applied values of low and high pressure were 0.28 and 414 MPa, respectively. A constant surface tension of 480 mN/m and a constant contact angle of 140° were assumed for the pore size calculation.

#### 2.3.5. Scanning Electron Microscopy

The microstructure of EUHPC was examined by scanning electron microscopy (SEM) (Zeiss, Jena, Germany). The patches taken from the compression test were fabricated into samples with a size less than 10 mm. The samples were soaked in 99.8% isopropyl alcohol to stop the hydration of cement. Thereafter, the samples were dried at 105 °C for 24 h and then coated with gold powder in the sputter coater before SEM imaging. 

## 3. Results and Discussion

### 3.1. Slump Flow

The flowabilities of UHPC mixtures are summarized in Table 3. It is clear that the flow diameter of all mixes varied between 180 cm and 260 cm. The mixtures of S20F0G0 with a small content of supplementary materials had the lowest spread of 180 mm. With the increase in supplementary material content, the flowability of EUHPC improved significantly. This behavior is in accordance with the results in [33]. For instance, when the content of supplementary materials, including SF, FA, and GGBFS, increased from 0.2 to 0.3, 0.4, and 0.5, the spread was increased by 13.9%, 36.1%, and 44.4%, respectively. It is important to note that the spread of mortar was greater than 240 mm when the proportion of supplementary materials exceeded 0.4, indicating that the EUHPC developed here meets the demand of self-leveling. Additionally, the comparisons of spread for the mixtures with different contents of FA and GGBFS indicate that the effect of fly ash on the EUHPC spread was better than that of GGBFS. For example, the spread of S20FA10G10 was higher than that of S20G20. The different results can be attributed to the effect of the particle shape of FA and GGBFS, whereby the former was spherical while the latter was angular. The sharp and angular particles would restrict the mortar adjacent to the GGBFS particle, resulting in insufficient lubrication to improve the flowability of fresh EUHPC.

### 3.2. Mechanical Properties 

#### 3.2.1. Mechanical Properties under Standard Curing 

**Figure 1:**
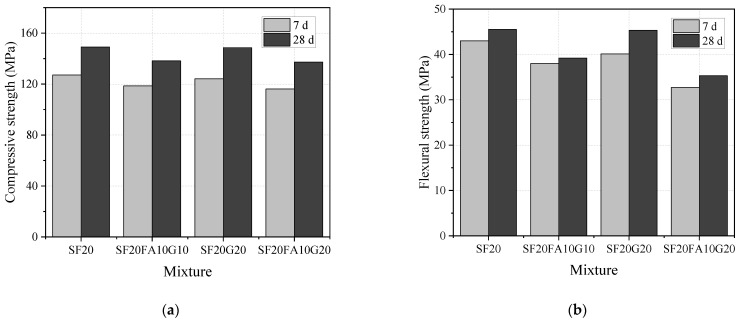
Compressive and flexural strengths of EUHPC under standard curing: (**a**) compressive strength; (**b**) flexural strength.

The compressive strength (*f*_c_) and flexural strength (*f*_b_) of EUHPC under standard curing are summarized in Table 3. It is clear that the reference mixture S20F0G0 without fly ash and slag under standard curing had the highest compressive strength and flexural strength, which decreased with the increase in fly ash and slag proportions. For instance, as the ratio of supplementary materials increased from 0.2 to 0.5, the 28 days compressive strength of EUHPC was decreased by 3.8%, 7.3%, and 7.9%, and the 28 days flexural strength was decreased by 0.4%, 13.8%, and 22.4%, respectively, compared with the reference mixture. This is attributed to the fact that the silica fume (SF) has a high pozzolanic effect even under a low curing temperature, which can react with calcium hydration (CH) to promote the hydration of cement. In addition, the incorporation of SF can fill the micro-pores of concrete due to the extremely small particle dimension and improve the interfacial transition zone (ITZ) of EUHPC, thus increasing the concrete strength. However, the pozzolanic effects of fly ash and GGBFS were difficult to stimulate at an early age under low curing temperature, thus resulting in lower strength of EUHPC with increasing fly ash and GGBFS content. Additionally, it can be inferred that the effect of supplementary materials on the flexural strength was more significant than that on the compressive strength. Furthermore, the effect of supplementary materials on the 7 days mechanical strength was weaker than that on the 28 days strength. This reason can be ascribed to the low hydration degree of supplementary materials at the early age. The strength comparisons of S20FA10G10, S20G20, and S20FA10G20 are presented in Figure 1. Both the 7 days and the 28 days compressive and flexural strengths of the mixture with 20% GGBFS were greater than that with 10% fly ash and 10% GGBFS, indicating that GGBFS had a smaller passive effect on the compressive and flexural strengths than fly ash. This is attributed to fact that the activity of fly ash at the early age was weak compared with GGBFS.

#### 3.2.2. Mechanical Properties under Steam Curing

**Figure 2:**
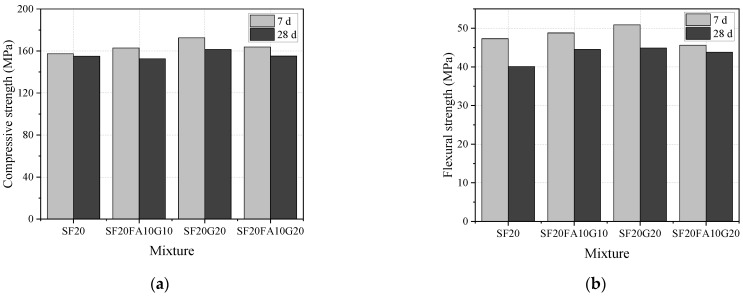
Compressive and flexural strengths of EUHPC under steam curing: (**a**) compressive strength; (**b**) flexural strength.

The compressive and flexural strengths of EUHPC under steam curing are summarized in Table 4 and Figure 2. The compressive strength of specimens under steam curing fell within the range of 151.8–161.5 MPa at the age of 28 days and within the range of 155.1–172.5 MPa at the age of 7 days. There was no significant increase in 28 days compressive strength for all mixtures cured in steam conditions compared with the strength at 7 days; inversely, a slight reduction in the compressive strength was observed for some mixtures. For instance, the 28 days compressive strengths were decreased by 1.4%, 6.3%, 6.4%, 5.9%, and 5.7% in comparison with those at 7 days, while the 28 days compressive strength of S20F10G10 under steam curing had a slight increase of about 0.8% compared with the 7 days strength. The decrease in compressive strength for S20F0G0 and S20F10G0 with low supplementary material content was small, while it was about 6% for the other mixtures with a content of supplementary materials within the range of 0.4 to 0.5. The high early strength was not conducive to the further development of strength, which can be ascribed to the expansion of concrete due to water absorption and the generation of ettringite under steam curing.

**Figure 3:**
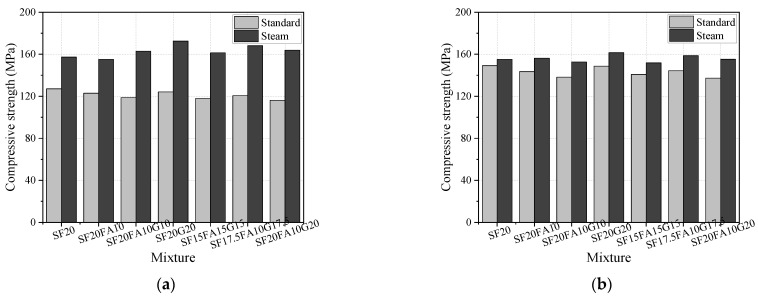

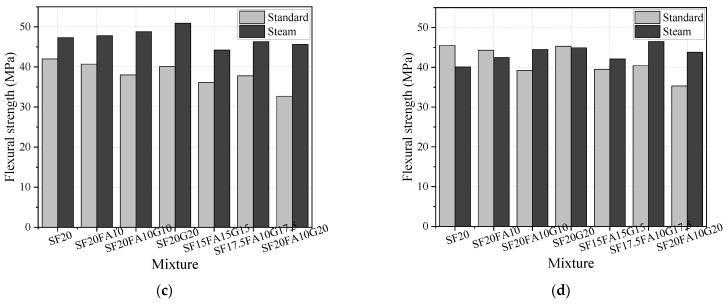
Compressive and flexural strength of EUHPC under different curing regimes: (**a**) compressive strength at 7 days; (**b**) compressive strength at 28 days; (**c**) flexural strength at 7 days; (**d**) flexural strength at 28 days.

The comparisons of the compressive and flexural strengths for EUHPC specimens under standard and steam curing are presented in Figure 3. The development of compressive strength at 7 days was fast under steam curing, whilst the mixtures with high supplementary material content had a higher compressive strength and flexural strength, as shown in Figure 3a. The mixtures S20F0G20 and S17.5F10G17.5 with a high content of GGBFS had greater increases in compressive strength (9.6% and 6.9%, respectively) in comparison with S20F0G0. This is because the activity of supplementary materials could be activated considerably due to the high temperature under steam curing; specifically, the GGBFS had higher activity compared with fly ash. Additionally, the 7 days compressive and flexural strengths under steam curing were significantly improved in comparison with standard curing, whereby the compressive strengths of S20F0G0, S20F10G0, S20F10G10, and S20F0G20 under steam curing were increased by 23.8%, 26.2%, 36.6%, and 39.0% compared with standard curing, respectively. Moreover, the increase in strength was more significant as the supplementary material content increased. However, the improvement in the 28 days compressive strength triggered by steam curing was not as significant as that in the 7 days compressive strength (4.0% to 13.0% vs. 23.8% to 41.1%). This is reasonable because the activity of supplementary materials could be stimulated under high temperature at early ages, and the compactness of the EUHPC matrix was reinforced due to the secondary reaction of the pozzolanic effect. The activities of fly ash and GGBFS and the pozzolanic effect were well developed after 28 days under standard curing but insignificant at the early ages. Furthermore, the flexural strength of the mixtures with a slight decrease in supplementary material content but increased distinctly for mixtures incorporated with abundant supplementary materials, showing no significant pattern.

### 3.3. XRD Analysis

#### 3.3.1. XRD Analysis of EUHPC under Standard Curing

**Figure 4:**
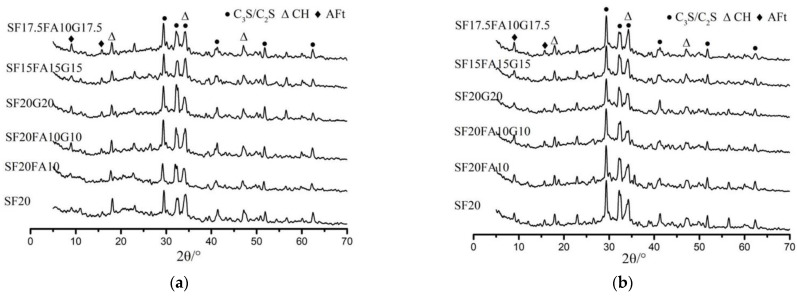
XRD of EUHPC at the age of 7 days and 28 days under standard curing: (**a**) XRD at the age of 7 days; (**b**) XRD at the age of 28 days.

The XRD spectra of EUHPC under standard curing at 7 days and 28 days are presented in Figure 4. It is clear that the diffraction peaks of C_3_S and C_2_S were significant for all specimens at 7 days and 28 days due to the relatively low water/binder ratio of EUHPC. Theoretically, the water demand for the complete hydration of cement is 23–25% of its weight, but many factors may influence the actual hydration of cement; the actual water requirement is about 42%, which is far higher than the theoretical value. The results of the hydration test of UHPC with the water/binder ratio of 0.16 indicated that the hydration degree of cement at 28 days was only 57.5%, with nearly half of the cement remaining unhydrated. Therefore, this is far from sufficient for the complete hydration of cement, and the pozzolanic effect of supplementary materials for the EUHPC with a water/binder ratio of 0.16 resulted in a low hydration degree of cement. The comparisons of the diffraction peaks of C_3_S and C_2_S at 7 days and 28 days under standard curing revealed slight variations at 28 days, indicating that the hydration of EUHPC continued slowly under standard curing due to the extremely low water/binder ratio.

It can be seen that the diffraction peaks of ettringite at 7 days became more obvious with the increase in supplementary material content, according to the comparison of S20F0G0, S20F10G0, and S20F10G10, whereas the diffraction peaks of ettringite were similar for the mixtures with similar supplementary material contents, as according to the comparison of S20F10G10, S20F0G20, S15F15G15, and S17.5F10G17.5. This may be related to the fact that the reaction of supplementary materials was not triggered at the early age, whereas the cement content decreased with the increase in supplementary materials content, whilst the water/binder ratio was constant, thus increasing the actual water–cement ratio; the free water could promote the hydration of cement to produce ettringite. Additionally, it can be seen from Figure 4b that ettringite was present for mixtures of S20F0G0–S17.5F10G17.5 after 28 days of curing and the diffraction peaks were similar, indicating that ettringite crystals could exist steadily in EUHPC under standard curing and the supplementary materials did not affect their formation.

Ca(OH)_2_ is an important index to assess the cement hydration degree. With the hydration of C_3_S and C_2_S in cement, a great mass of Ca(OH)_2_ is generated at the early age, indicating that the cement has achieved a great hydration degree. It should be emphasized that the Ca(OH)_2_ diffraction peaks in the six mixtures were very similar at the age of 7 days, because the free water needed to promote the cement hydration reaction to produce more Ca(OH)_2_ as the secondary material increased under rising water-to-ash ratio, whereas some Ca(OH)_2_ could continue hydration to produce C–S–H gel, resulting in six mixtures with similar crystallinity. However, the Ca(OH)_2_ diffraction peaks at the age of 28 days were decreased compared with those at 7 days, which is reasonable because the pozzolanic effects of silica fume, fly ash, and GGBFS consumed Ca(OH)_2_ in the EUHPC and produced C–S–H gel to further compact the matrix structure. Thus, a higher compressive strength could be attained for EUHPC with high supplementary material content.

#### 3.3.2. XRD Analysis of EUHPC under Steam Curing

**Figure 5:**
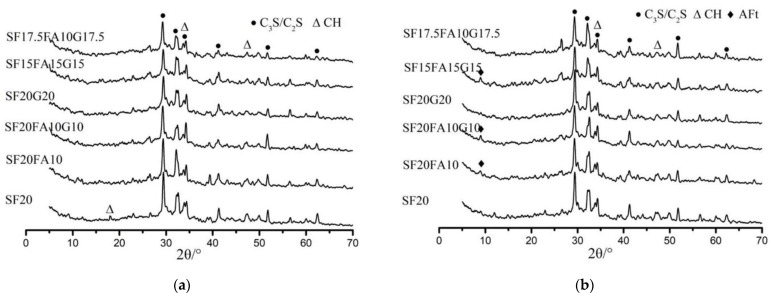
XRD of EUHPC at the age of 7 days and 28 days under steam curing: (**a**) XRD at the age of 7 days; (**b**) XRD at the age of 28 days.

The XRD figures at 7 days and 28 days of EUHPC under steam curing are plotted in Figure 5. As can be seen from the figures, the diffraction peaks of C_3_S and C_2_S of EUHPC were still the most significant even after the steam curing. It should be noted that the diffraction peaks of C_3_S and C_2_S at 7 days and 28 days were nearly identical. This is attributed to the fact that steam curing could promote the hydration of C_3_S and C_2_S in cement; as the free water was nearly exhausted in the first 7 days, the cement hydration was almost at a standstill after 7 days, partially contributing to the decline in 28 days strength compared with the 7 days strength.

The diffraction peaks of ettringite for EUHPC after steam curing exhibited significant changes, whereby they almost disappeared compared with standard curing. This is because the ettringite produced by cement hydration was decomposed under high temperature or translated into single-sulfur calcium sulfoaluminate hydrate (AFm), which could not be detected by XRD. Furthermore, ettringite causes the expansion of set concrete and results in cracking and pores in the matrix, whereas AFm can compact the matrix, resulting in defects in the pore structure at the later ages of EUHPC, which was the major reason for the decrease in 28 days compressive strength. Figure 4b shows that the diffraction peaks of ettringite for some mixtures at the age of 28 days appeared in the plot, which were nonexistent in the 7 days XRD figure. It can be inferred that the ettringite generated at the early ages had low stability, later recrystallizing in a suitable space after the decomposition induced by high temperature with the migration of solution.

EUHPC under steam curing had a lower diffraction peak intensity of Ca(OH)_2_ in comparison with standard curing, which was primarily induced by the consumption of Ca(OH)_2_ via the pozzolanic effect of supplementary materials. This indicates that there was a dramatic pozzolanic effect in the first 7 days, whereby the high temperature under steam curing not only promoted the hydration rate of cement but also improved the pozzolanic effect of supplementary materials, and the fine pores of EUHPC could be filled by the formed C–S–H gel. The Ca(OH)_2_ diffraction peak intensity of S20F0G0 in Figure 5a was more remarkable than that of other mixtures, since the silica fume could react with Ca(OH)_2_, thus reducing its content. However, the content of silica fume was limited; hence, the content of Ca(OH)_2_ was still higher than in other mixtures. Furthermore, the pozzolanic effect of fly ash and GGBFS could react with Ca(OH)_2_ and further reduce its content. The diffraction peaks of Ca(OH)_2_ in the mixtures of S20FA10–SF17.5FA10G17.5 were weakened to a certain degree, demonstrating that the significant pozzolanic effect of composite supplementary materials in EUHPC was remarkable under steam curing, generating a large amount of amorphous C–S–H gel to further compact the microstructure of the matrix.

### 3.4. MIP Analysis 

**Figure 6:**
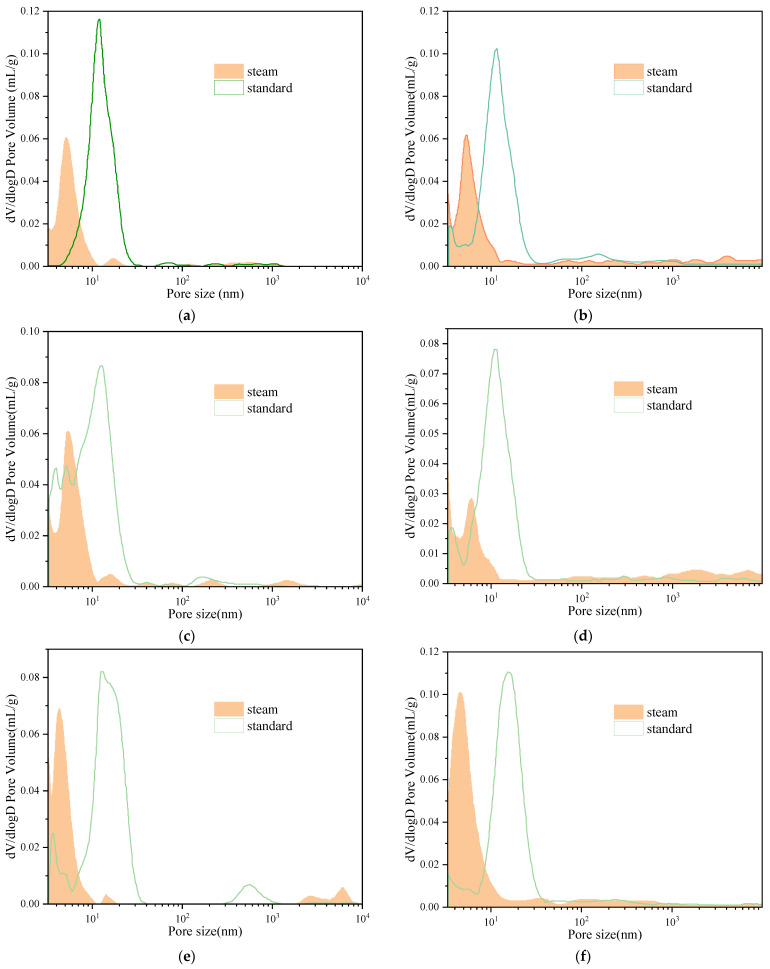
Pore size distribution of EUHPC samples under standard and steam curing: (**a**) S20F0G0; (**b**) S20F10G0; (**c**) S20F10G10; (**d**) S20F0G20; (**e**) S15F15G15; (**f**) S20F0G0*.

The pore structure is a key factor to determine the mechanical properties and durability of concrete, which can be characterized by mercury intrusion porosimetry (MIP). However, the noncontinuity, closure, and inkstand effect of the pore structure can affect the effectiveness of the method. The test results of MIP were sufficient to characterize the pore differences for the EUHPC specimen under different curing regimes. Since the cement hydration of EUHPC was mainly performed in the first 7 days before developing slowly, the samples for the MIP test were taken from the specimens after curing for 7 days. Figure 6 gives the pore size distribution of the EUHPC samples under standard and steam curing. The pore size was lower than 30 nm under standard curing, while it was below 10 nm under steam curing. This can be ascribed to the fact that the cement hydration rate could be improved by steam curing and the pozzolanic effect of composite supplementary materials could be fully developed, further compacting the microstructure of the matrix.

The comparisons from Figure 6a–c show that the micropore structure distribution curves of the mixtures S20F10G0 and S20F10G10 under steam curing were identical to those of S20F0G0 with the lowest supplementary materials. However, the differential pore volume under standard curing decreased with the increase in supplementary material content. Since the pozzolanic effect of supplementary materials was not significant at the age of 7 days under standard curing, the micro-aggregate effect was prominent. The comparisons from Figure 6c–e signify that the micropore volume of S20F0G20 was similar to that of S20F10G10 and S15F15G15 under standard curing, while the micropore volumes under steam curing were minimal. This was due to the high pozzolanic effect of silica fume and GGBFS, which had higher reactivity and induced a denser matrix of EUHPC.

The pore structures of EUHPC under standard curing are summarized in Table 5. The mixture of S20F0G0* without defoamer was excluded from the comparisons. According to the classification, a pore size<10 nm is microcapillary, which is favorable to the improvement of durability, a pore size<20 nm is harmless, a pore size of 20–50 nm is less harmful, a pore size of 50–200 nm is harmful, and a pore size greater than 200 nm is more harmful. The porosity of EUHPC under standard curing varied between 7% and 9%, with the most probable pore size of about 10–12 nm; the pore size distribution was mainly below 20 nm and prominent in the range of 10–20 nm. The porosities of the mixtures S20FA10 and S20F10G10 were higher than that of S20F0G0, which was not reduced upon the incorporation of fly ash. This can be ascribed to the large particle size of fly ash, whose activity is weak with a prominent micro-aggregate effect at the early ages. The porosities of the mixtures S20F0G20 and S20F10G10 were slightly lower than that of S20F0G0, demonstrating that the high activity and the micro-aggregate effect of GGBFS were beneficial to improving the pore structure of EUHPC.

The pore structures of EUHPC under steam curing are summarized in Table 6. The porosity of EUHPC under steam curing ranged between 3.5% and 5%, with a pore size mainly below 20 nm, in addition to the prominence of microcapillary pores with a size smaller than 10 nm, which was favorable to the durability of EUHPC. The porosities of S20F10G0, S20F10G10, and S20F0G20 under steam curing were higher than that of S20F0G0, indicating the fly ash could not significantly reduce the porosity of EUHPC. In addition, the porosity of S20F0G20 was 3.51%, which was far lower than that of the other mixtures; this could be attributed to the joint activation of silica fume and GGBFS under steam curing, where the pozzolanic effect consumed Ca(OH)_2_ and produced C–S–H to further compact the matrix.

The comparisons from Table 5 and Table 6 show that the total pore volumes of S20F10G0–S17.5F10G17.5 under steam curing were decreased by 45.5%, 38.9%, 51.2%, 49.3%, and 42.3% compared with those under standard curing, the corresponding most probable pore sizes were decreased by 56.8%, 49.1%, 51.3%, 51.5%, and 64%, respectively. Hence, it can be inferred that steam curing could significantly promote the hydration rate of EUHPC and the pozzolanic effect of supplementary materials in the early ages, as reflected in the macroscopic strength.

The effect of defoamer on the pore structure of EUHPC is also discussed according to the asymmetric mixture design S20*F0G0 without defoamer. It can be seen from Table 7 that the porosities of S20*F0G0 under standard curing and steam curing were higher than those of S20F0G0. The most probable pore size of S20F0G0* under standard curing was 20.7 nm, located in the range of less harmful pores, which was much larger than that of S20F0G0. On the other hand, the most probable pore sizes of S20*F0G0 and S20F0G0 under steam curing were similar. This can be attributed fact that the defoamer could decrease the surface tension inside the bubbles of cement mortar and reduce the number of pores, thus optimizing the pore structure and improving the quality of concrete. It is worth noting that the cement hydration and the pozzolanic effect of supplementary materials of EUHPC were well developed under steam curing, the number of pores was decreased dramatically, and the microstructure was improved significantly; hence, the effect of defoamer on the pore size and porosity became weak. The proportion of harmful pores increased significantly compared with the mixtures with defoamer, indicating that the deformer significantly improved the pore structure of EUHPC.

### 3.5. SEM Analysis

**Figure 7:**
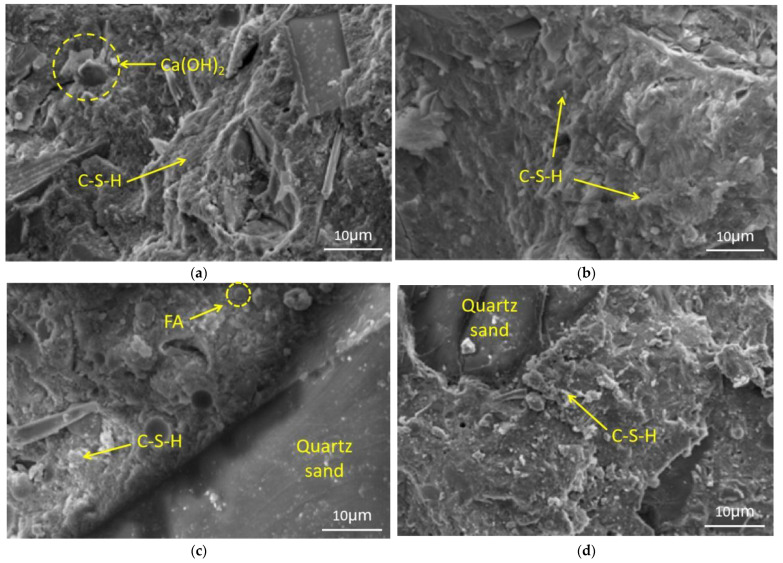
SEM images of EUHPC under different curing regimes: (**a**) S20F0G0 under standard curing; (**b**) S20F0G0 under steam curing; (**c**) S20F10G10 under standard curing; (**d**) S20F10G10 under steam curing.

**Figure 8:**
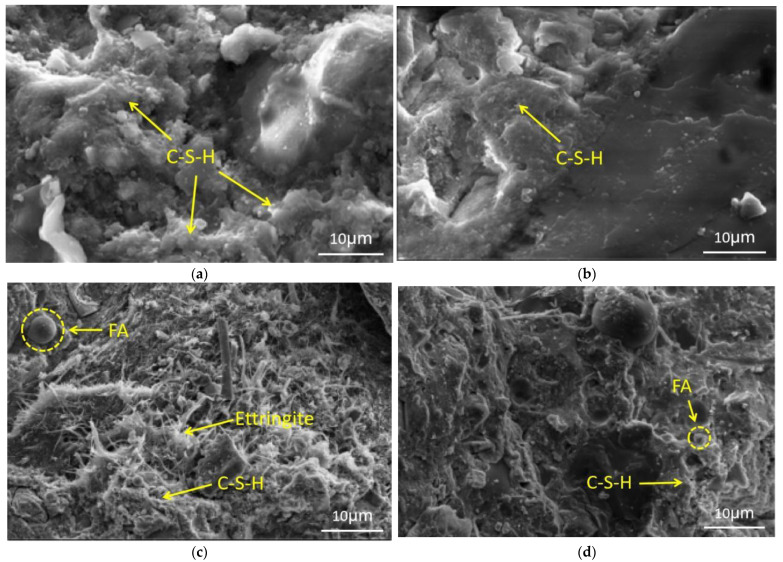
SEM images of EUHPC under standard curing and steam curing: (**a**) S20F0G20 under standard curing; (**b**) S20F0G20 under steam curing; (**c**) S15F15G15 under standard curing; (**d**) S15F15G15 under steam curing.

Scanning electron microscopy (SEM) is conducted to study the microstructure and morphology of EUHPC under standard and steam curing after 7 days. Taking S20F0G0 and S20F10G10 as examples, the SEM images of EUHPC under different curing conditions are presented in Figure 7 and Figure 8. It can be seen from Figure 7a (reference sample) that Ca(OH)_2_ and C–S–H gel can be clearly observed in the set cement of S20F0G0 under standard curing. On the other hand, in the SEM images of S20F0G0 under steam curing, many C–S–H gels grew interlaced with each other, while Ca(OH)_2_ was hardly observed, indicating that the microstructure of EUHPC under steam curing was much denser than that under standard curing. Figure 7c presents the SEM image of S20F10G10 under standard curing, where unhydrated fly ash and GGBFS particles can be observed in the microstructure, as well as the fusiform C–S–H gel in the growth process. In addition, the combination of matrix and the angular quartz sand was close, whereby the interfacial transition zone (ITZ) was almost invisible. The SEM image of S20F10G10 under steam curing is shown in Figure 7d, where no fly ash and GGBFS particles can be observed in the matrix. It can be inferred that the pozzolanic effects of fly ash and GGBFS were sufficient under steam curing. The comparison of SEM images between S20F0G0 and S20F10G10 indicates that the addition of fly ash and GGBFS could significantly improve the compactness of matrix microstructure due to the pozzolanic effect of supplementary materials promoting the formation of C–S–H.

The microstructures of S20F0G20 under standard curing and steam curing are presented in Figure 8. No unhydrated GGBFS particles could be observed in the matrix of S20F0G20, while the patchy C–S–H gel was visible. Additionally, the hydration reaction degree was higher than that of S20F10G10, and the microstructure of the S20F0G20 matrix was much denser, indicating that GGBFS had higher activity than fly ash. The mixture S20F0G20 incorporated with silica fume and GGBFS under standard and steam curing had higher reactivity compared with the other mixtures, where the high activity of GGBFS was beneficial to the formation of a dense matrix. Figure 8c presents the SEM image of S15F15G15 under standard curing, in which unhydrated fly ash and GGBFS particles, as well as Ca(OH)_2_, can be observed in the figure, and many acicular ettringite crystals filled the hardened matrix, signifying that the EUHPC was in the process of rapid hydration. The SEM image of S15F15G15 under steam curing is shown in Figure 8d, where it can be observed that the set cement was mainly composed of amorphous C–S–H gel, while some unhydrated fly ash and GGBFS particles were still embedded in the hardened matrix; thus, the microstructure under steam curing was more compact than that under standard curing.

## 4. Conclusions

The effect of curing regime on the mechanical strength, hydration, pore size distribution, and microstructure of EUHPC was studied experimentally in this work. The content of supplementary materials, including silica fume, fly ash, and ground granulated blast furnace slag was considered. On the basis of the results of this study, the following conclusions can be drawn:

(1) According to the results of flowability tests and mechanical properties, the mixtures S20F0G20 and S17.5F10G17.5 had the best overall performance. The 28 days compressive strength of both mixtures under standard curing exceeded 140 MPa, while the flexural strength exceeded 40 MPa. The flowabilities of the two mixtures were similar, while S17.5F10G17.5 had a lower viscosity, which is beneficial for improving the workability of EUHPC.

(2) Steam curing improved the early strength, whereby the 7 days compressive strength of S20F0G20 under steam curing reached 172.5 MPa, while the 7 days flexural strength attained 50.9 MPa. The strength decreased with age under steam curing; the decrease in compressive strength for EUHPC with a high content of supplementary materials was about 6%.

(3) Substantial amounts of C_3_S and C_2_S were observed in the hardened EUHPC under standard curing and steam curing, as much of the cement was unhydrated. The hydration degree of EUHPC under steam curing was higher than that under standard curing at the age of 7 days, while the pozzolanic effect was more significant under steam curing. After 7 days, the reaction of EUHPC under standard curing accelerated gradually, accompanied by the pozzolanic effect of supplementary materials, while the hydration of EUHPC and the pozzolanic effect under steam curing almost stopped after 7 days.

(4) The pore size distribution of EUHPC under standard curing was mainly below 20 nm. The porosity under standard curing ranged between 7% and 9%, and the pore size was mainly in the range of 10–20 mm, while the porosity of EUHPC under steam curing is 3.5–5%, and microcapillary pores with a size less than 10 nm were dominant.

(5) Unhydrated fly ash, GGBFS particles, and Ca(OH)_2_ crystals were observed in the amorphous C–S–H gel under standard curing. A large number of C–S–H gels grew interlaced with each other, and it was difficult to observe the existence of Ca(OH)_2_ crystals in the hardened matrix of EUHPC under steam curing. The microstructure of the sample under steam curing was denser than that of under standard curing, whereas the interfacial transition zones (ITZ) of EUHPC under both standard and steam curing were extremely compact.

Generally, steam curing could significantly improve the early strength of EUHPC, while the increase in 28 days strength was neither significant nor stable. Hence, steam curing can be utilized in practical engineering with high requirements for early strength. Due to the complicated construction and additional cost of steam curing, it is not recommended for normal EUHPC engineering, as standard curing is able to meet the needs of practical engineering. To reduce the content of cement and enhance the overall performance in UHPC, the dosage of supplementary materials in EUHPC is recommended to be 40% by mass fraction of cementitious materials, with a ratio of silica fume, fly ash, and GGBFS of about 3:2:3. Furthermore, the effects of curing regime and ratio of supplementary materials on the durability and the elevated temperature properties of EUHPC need to be further studied.

## Figures and Tables

**Table 1:** Chemical components of cement, silica fume, fly ash, and slag (%).

Chemical Components	MgO	Al_2_O_3_	SiO_2_	Fe_2_O_3_	CaO	K_2_O	Na_2_O	SO_3_	P_2_O_5_
Cement	2.14	4.5	19.58	3.119	64.94	0.75	0.079	3.06	0.128
Silica fume (SF)	0.224	0.354	92.87	0.113	0.213	0.332	0.068	1.26	0.11
Fly ash (FA)	0.575	30.63	48.74	2.611	2.44	1.25	0.552	0.706	0.247
Slag (GGBFS)	9.46	11.53	36.39	0.218	36.79	1.56	0.22	0.84	--

**Table 2:** Mix proportions of EUHPC.

Mixtures	Cementitious Materials				Quartz Sand	W/B Ratio	Defoamer
	Cement	SF	FA	GGBFS			
S20F0G0	0.80	0.20			1.0	0.16	0.1%
S20F10G0	0.70	0.20	0.10		1.0	0.16	0.1%
S20F10G10	0.60	0.20	0.10	0.10	1.0	0.16	0.1%
S20F0G20	0.60	0.20	0	0.20	1.0	0.16	0.1%
S15F15G15	0.55	0.15	0.15	0.15	1.0	0.16	0.1%
S17.5F10G17.5	0.55	0.175	0.10	0.175	1.0	0.16	0.1%
S20F10G20	0.50	0.20	0.10	0.20	1.0	0.16	0.1%
S20*F0G0	0.80	0.20			1.0	0.16	0

Note: The total weight of cementitious materials was set as 1; “S” is for “SF”, “F” is for “FA”, “G” is for “GGBFS”; the proportion of each component is denoted as the ratio by the total weight of cementitious materials; * represents that the defoamer was removed in the mixture.

**Table 3:** Flowability and mechanical properties of EUHPC under standard curing.

Specimen	Slump Flow (mm)	7 Days (MPa)		28 Days (MPa)	
		*f*_c_	*f*_b_	*f*_c_	*f*_b_
S20F0G0	180	127.1	42.0	149.1	45.5
S20F10G0	205	122.9	40.7	143.4	44.3
S20F10G10	245	118.6	38.0	138.2	39.2
S20F0G20	240	124.1	40.1	148.6	45.3
S15F15G15	255	117.8	36.1	140.8	39.5
S17.5F10G17.5	255	120.5	37.8	144.3	40.4
S20F10G20	260	116.1	32.7	137.3	35.3

**Table 4:** Mechanical properties of EUHPC under steam curing.

Specimen	7 Days (MPa)		28 Days (MPa)	
	*f*_c_	*f*_b_	*f*_c_	*f*_b_
S20F0G0	157.3	47.3	155.1	40.1
S20F10G0	155.1	47.8	156.3	42.5
S20F10G10	162.9	48.8	152.6	44.5
S20F0G20	172.5	50.9	161.5	44.9
S15F15G15	161.3	44.2	151.8	42.1
S17.5F10G17.5	168.1	46.3	158.6	46.5
S20F10G20	163.8	45.6	155.2	43.8

**Table 5:** The pore structure of EUHPC under standard curing.

Specimen	Probable Pore (nm)	Porosity (%)	Pore Size Distribution				
			<10 nm	10–20 nm	20–50 nm	50–200 nm	>200 nm
S20F0G0	12.5	7.86	15.56	75.25	3.61	1.66	3.92
S20F10G0	10.8	8.00	30.15	59.34	4.73	2.31	3.47
S20F10G10	11.3	9.31	42.16	46.33	1.79	4.49	5.23
S20F0G20	9.9	6.92	37.89	54.82	2.20	0.83	4.27
S15F15G15	12.5	7.80	29.65	54.89	10.66	0.96	4.80

**Table 6:** The pore structure of EUHPC under steam curing.

Specimen	Probable Pore (nm)	Porosity (%)	Pore Size Distribution				
			<10 nm	10–20 nm	20–50 nm	50–200 nm	>200 nm
S20F0G0	5.4	4.28	70.90	24.82	0.20	0.41	3.67
S20F10G0	5.5	4.89	75.29	20.54	1.06	1.31	1.80
S20F10G10	5.5	4.77	69.82	20.66	0.82	0.64	3.06
S20F0G20	4.8	3.51	79.61	15.55	1.00	1.20	2.64
S15F15G15	4.5	4.50	82.85	8.68	3.72	0.61	4.14

**Table 7:** The pore structure of S20F0G0* without defoamer under standard curing and steam curing.

Curing Agent	Probable Pore (nm)	Porosity (%)	Pore Size Distribution				
			<10 nm	10–20 nm	20–50 nm	50–200 nm	>200 nm
Standard curing	20.7	10.18	13.20	62.87	15.56	1.87	6.49
Steam curing	5.6	6.31	80.36	11.89	1.83	2.00	3.91

## Data Availability

The data used to support the findings of this study can be made available from the corresponding author upon request.
